# Influence of marital status on the survival of adults with extrahepatic/intrahepatic cholangiocarcinoma

**DOI:** 10.18632/oncotarget.16330

**Published:** 2017-03-17

**Authors:** Zhiqiang Chen, Liyong Pu, Wen Gao, Long Zhang, Guoyong Han, Qin Zhu, Xiangcheng Li, Jindao Wu, Xuehao Wang

**Affiliations:** ^1^ Department of Liver Surgery, The First Affiliated Hospital of Nanjing Medical University, Key Laboratory on Living Donor Liver Transplantation, National Health and Family Planning Commission, Nanjing, Jiangsu Province, China; ^2^ Department of Oncology, The First Affiliated Hospital of Nanjing Medical University, Nanjing, Jiangsu Province, China

**Keywords:** cholangiocarcinoma, marital status, SEER, survival analysis, prognosis

## Abstract

Although the prognostic value of marital status has been implicated in many cancers, its prognostic impact on cholangiocarcinoma has not yet been determined. The aim of this study was to examine the association between marital status and cholangiocarcinoma survival. We included 8,776 extrahepatic cholangiocarcinoma cases and 1,352 intrahepatic cholangiocarcinoma cases between 1973 and 2013 from the Surveillance, Epidemiology, and End Results database. We found widowed patients were more likely to be female, aged more than 70, and from low income areas. Multivariate analysis indicated that marital status was an independent prognostic factor for extrahepatic cholangiocarcinoma patients. Subgroup analysis suggested the widowed status independently predicted poor survival at regional stage and in older patients with intrahepatic cholangiocarcinoma. To conclude, marital status is a valuable prognostic factor in cholangiocarcinoma, and widowed patients are at greater risk of death than others.

## INTRODUCTION

Cholangiocarcinoma, the second most common primary hepatic cancer after hepatocellular carcinoma (HCC), accounts for approximately 3% of all gastrointestinal malignancies [1, 2]. Cholangiocarcinoma are anatomically classified as extrahepatic cholangiocarcinoma (ECC) or intrahepatic cholangiocarcinoma (ICC) according to its location with regard to the liver [3]. The incidence of ECC/ICC has increased substantially in the past decades, and the newly diagnosed cases have exceeded 7,000 annually [4, 5]. Both ECC and ICC are highly lethal and characterized by an aggressive behavior with early lymphatic spread and distant metastasis. Total cholangiocarcinoma mortality for those aged more than 25 increased 36% between 1999 and 2014, from 2.2 per 100,000 to 3.0 per 100,000 [6, 7].

An emerging number of studies have demonstrated the involvement of marriage in the clinical prognosis of various digestive system malignancies. Among pancreatic cancer patients, marital status was an independent prognostic factor [8]. In patients with colorectal neuroendocrine neoplasms, the widowed group were at greater risk of cancer specific mortality [9]. Reportedly, widowed patients suffered from the poorest 5-year cancer specific survival in HCC [10]. In primary liver cancer patients, widowed patients had a survival disadvantage while married persons enjoyed survival benefits in both cancer-specific survival and overall survival [11]. Paradoxically, a cross-sectional study conducted in wetland communities of Ubon Ratchathani in Thailand implied that married participants had a 2.61 times higher risk of cholangiocarcinoma than unmarried participants [12].

In order to clarify the prognostic significance of marital status in cholangiocarcinoma, we performed a comprehensive population-based analysis. The data were obtained from the Surveillance, Epidemiology, and the End Results (SEER) cancer registry, which comprised about 97% of incident cancer cases from 17 cancer registries representing 28% of the US population. Due to the differences in pathogenesis, etiologic risk factors, and genetic characteristics between ECC and ICC [13–18], ECC cause-specific survival (ECSS) and ICC cause-specific survival (ICSS) were examined separately in our analysis.

## RESULTS

### Baseline patient characteristics

A total of 10,128 eligible cholangiocarcinoma patients were included during the 40-year study period (from 1973 to 2013), comprising 5,265 male and 4,863 female patients. Among these patients, 5,926 (58.5%) were married, 1,150 (11.4%) had never married, 947 (9.4%) were divorced/separated and 2,105 (20.8%) were widowed. Significant differences were observed in all subgroups, including gender, age, ethnicity, year of diagnosis, pathological grading, TNM stage, SEER stage and socioeconomic status. All comparisons were statistically significant (*P* < 0.001). Of note, married patients were more likely to be male (63.2%), while widowed patients have the highest proportion (79.4%) of female patients. Widowed patients also had a greater proportion (82.5%) of older patients. Additionally, the socioeconomic status of married individuals was better than other unmarried individuals. Married patients had the largest proportion of low poverty (16.3%), and suffered the least from high poverty (15.4%). The baseline cholangiocarcinoma patient demographics and malignancy characteristics were summarized in Table 1.

**Table 1:** Baseline demographic and cancer characteristics of cholangiocarcinoma patients in SEER database

Characteristic	Total	Married	Never married	Divorced/Separated	Widowed	*P*
	(*n* = 10128) N(%)	(*n* = 5926) N(%)	(*n* = 1150) N(%)	(*n* = 947) N(%)	(*n* = 2105) N(%)	
Gender						< 0.001
Male	5265(52.0)	3744(63.2)	629(54.7)	458(48.4)	434(20.6)	
Female	4863(48.0)	2182(36.8)	521(45.3)	489(51.6)	1671(79.4)	
Age						< 0.001
<70	5059(50.0)	3332(56.2)	782(68.0)	577(60.9)	368(17.5)	
≥70	5069(50.0)	2594(43.8)	368(32.0)	370(39.1)	1737(82.5)	
Ethnicity						< 0.001
White	7969(78.7)	4725(79.7)	828(72.0)	758(80.0)	1658(78.8)	
Black	835(8.2)	337(5.7)	186(16.2)	111(11.7)	201(9.5)	
Other*	1324(13.1)	864(14.6)	136(11.8)	78(8.2)	246(11.7)	
Year of diagnosis						< 0.001
1973–1979	496(4.9)	341(5.8)	19(1.7)	52(5.5)	84(4.0)	
1980–1989	785(7.8)	445(7.5)	69(6.0)	80(8.4)	191(9.1)	
1990–1999	1149(11.3)	670(11.3)	131(11.4)	87(9.2)	261(12.4)	
2000–2009	5074(50.1)	2911(49.1)	584(50.8)	468(49.4)	1111(52.8)	
2010–2013	2624(25.9)	1559(26.3)	347(30.2)	260(27.5)	458(21.8)	
Pathological grading						< 0.001
Well/Moderate	2826(27.9)	1839(31.0)	308(26.8)	251(26.5)	428(20.3)	
Poor/Anaplastic	1670(16.5)	1044(17.6)	173(15.0)	180(19.0)	273(13.0)	
Unknown	5632(55.6)	3043(51.3)	669(58.2)	516(54.5)	1404(66.7)	
TNM Stage						< 0.001
I/II	2664(26.3)	1614(27.2)	306(26.6)	263(27.8)	481(22.9)	
III/IV	2256(22.3)	1357(22.9)	306(26.6)	245(25.9)	348(16.5)	
Unknown	5208(51.4)	2955(49.9)	538(46.8)	439(46.4)	1276(60.6)	
SEER Stage						< 0.001
Localized	2181(21.5)	1250(21.1)	230(20.0)	202(21.3)	499(23.7)	
Regional	3978(39.3)	2504(42.3)	448(39.0)	359(37.9)	667(31.7)	
Distant	2445(24.1)	1476(24.9)	299(26.0)	253(26.7)	417(19.8)	
Unstaged	1524(15.0)	696(11.7)	173(15.0)	133(14.0)	522(24.8)	
Socioeconomic Status						< 0.001
Low poverty	1570(15.5)	966(16.3)	146(12.7)	139(14.7)	319(15.2)	
Medium poverty	6868(67.8)	4048(68.3)	806(70.1)	651(68.7)	1363(64.8)	
High poverty	1690(16.7)	912(15.4)	198(17.2)	157(16.6)	423(20.1)	

Abbreviations: SEER, Surveillance, Epidemiology, and End Results.

*Other includes American Indian/Alaska native, Asian/Pacific Islander, and unknown.

After further analyzing these differences in ECC and ICC respectively, as shown in Supplementary Table 1 and Supplementary Table 2, these differences were also observed in ECC patients. In ICC patients, by contrast, only gender, age, ethnicity, TNM stage and SEER stage remained significant.

### Influence of marital status on ECSS

**Figure 1:**
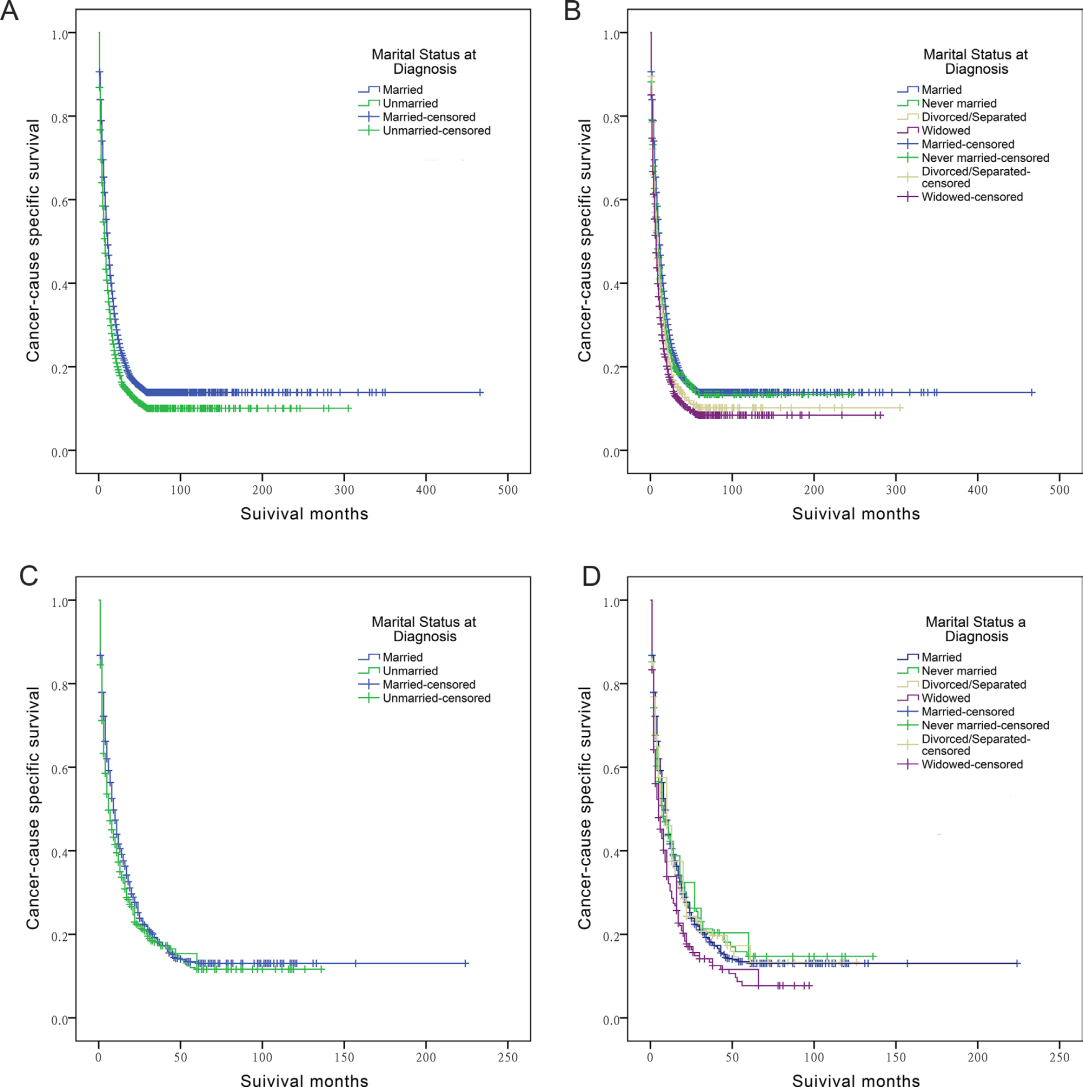
Survival curves in cholangiocarcinoma patients (**A**) The survival curve between married and unmarried ECC patients: χ^2^ = 104.720 (*P* < 0.001); (**B**) The survival curve between married, never married, divorced/separated, and widowed ECC patients: χ^2^ = 138.625 (*P* < 0.001); (**C**) The survival curve between married and unmarried ICC patients: χ^2^ = 3.646 (*P* = 0.056); (**D**) The survival curve between married, never married, divorced/separated, and widowed ICC patients: χ^2^ = 12.351 (*P* = 0.006).

Married individuals had a better 5-year ECSS than unmarried individuals (13.9% vs 10.1%) (*P* < 0.001) (Figure 1A). Specifically, the 5-year ECSS was 13.4% in the never married group, 10.2% in the divorced/separated group and 8.4% in the widowed group (Figure 1B). Female (*P* < 0.001), older age (*P* < 0.001), black race (*P* = 0.005), the latest year of diagnosis (*P* < 0.001), poor/anaplastic pathological grading (*P* < 0.001), TNM stage III/ IV (*P* < 0.001), SEER distant stage (*P* < 0.001) and high poverty (*P* < 0.001) were regarded as significant risk factors for a poorer survival by univariate analysis (Table 2). Additionally, multivariate analysis was performed by the Cox regression model. The following seven factors were verified as independent prognostic factors for ECC (Table 2), including age (≥70, hazard ratio [HR] 1.362, 95% confidence interval [CI] 1.293-1.436), year of diagnosis (1973-1979, HR 0.839, 95% CI 0.737-0.955; 1980-1989, HR 0.777, 95% CI 0.688-0.877; 1990-1999, HR 0.810, 95% CI 0.724-0.906; 2000-2009, HR 0.756, 95% CI 0.666-0.859), pathological grade (poor/ anaplastic, HR 1.480, 95% CI 1.369-1.599), TNM stage (III/ IV, HR 1.135, 95% CI 1.035-1.244), SEER stage (regional, HR 1.157, 95% CI 1.079-1.241; distant, HR 2.225, 95% CI 2.034-2.435), socioeconomic status (medium poverty, HR 1.074, 95% CI 1.004-1.149; high poverty, HR 1.191, 95% CI 1.094-1.297), and marital status (never married, HR 1.150, 95% CI 1.061-1.247; divorced/separated, HR 1.183, 95% CI 1.087-1.287; widowed, HR 1.179, 95% CI 1.104-1.260). However, no statistical differences were found with regard to gender and ethnicity according to multivariate survival analysis.

**Table 2:** Univariate and multivariate survival analysis for evaluating the influence of marital status on extrahepatic/intrahepatic cholangiocarcinoma cause-specific survival in SEER database

	Extrahepatic cholangiocarcinoma					Intrahepatic cholangiocarcinoma				
Variable		Univariate analysis		Multivariate analysis			Univariate analysis		Multivariate analysis	
	5-year CCS	Log rank χ2 test	*P*	HR (95% CI)	*P*	5-year CCS	Log rank χ2 test	*P*	HR (95% CI)	*P*
Gender		57.597	< 0.001		0.098		5.841	0.016		0.002
Male	13.7%			Reference		11.6%			Reference	
Female	10.8%			1.044(0.992–1.099)		13.4%			0.816(0.719–0.927)	
Age		192.854	< 0.001		< 0.001		13.549	< 0.001		< 0.001
<70	15.4%			Reference		14.1%			Reference	
≥70	9.4%			1.362(1.293–1.436)		9.7%			1.276(1.114–1.461)	
Ethnicity		10.477	0.005		NI		1.563	0.458		NI
White	12.3%					11.8%				
Black	10.2%					10.1%				
Other*	13.4%					21.3%				
Year of diagnosis		27.524	< 0.001		< 0.001		42.004	< 0.001		0.007
1973–1979	8.8%			Reference		4.2%			Reference	
1980–1989	10.6%			0.839(0.737–0.955)	0.008	3.7%			1.090(0.755–1.574)	0.645
1990–1999	13.1%			0.777(0.688–0.877)	< 0.001	10.0%			0.635(0.423–0.952)	0.028
2000–2009	12.5%			0.810(0.724–0.906)	< 0.001	13.9%			0.748(0.542–1.031)	0.076
2010–2013	#			0.756(0.666–0.859)	< 0.001	/			0.813(0.571–1.156)	0.249
Pathological grading		622.853	< 0.001		< 0.001		90.577	< 0.001		< 0.001
Well/ Moderate	22.5%			Reference		22.3%			Reference	
Poor/ Anaplastic	11.9%			1.480(1.369–1.599)	< 0.001	9.4%			1.540(1.282–1.850)	< 0.001
Unknown	6.9%			1.679(1.577–1.787)	< 0.001	8.5%			1.683(1.441–1.966)	< 0.001
TNM Stage		407.369	< 0.001		< 0.001		92.752	< 0.001		< 0.001
I/II	20.1%			Reference		30.0%			Reference	
III/ IV	4.4%			1.135(1.035–1.244)	0.007	6.2%			1.535(1.217–1.938)	< 0.001
Unknown	11.3%			1.194(1.104–1.290)	< 0.001	9.5%			1.704(1.371–2.118)	< 0.001
SEER Stage		943.197	< 0.001		< 0.001		131.019	< 0.001		< 0.001
Localized	18.9%			Reference		21.9%			Reference	
Regional	15.6%			1.157(1.079–1.241)	< 0.001	9.5%			1.236(1.025–1.491)	0.027
Distant	2.6%			2.225(2.034–2.435)	< 0.001	3.7%			1.852(1.523–2.252)	< 0.001
Unstaged	9.5%			1.208(1.103–1.323)	< 0.001	14.6%			1.107(0.876–1.400)	0.395
Socioeconomic Status		17.045	< 0.001		< 0.001		9.589	0.008		0.027
Low poverty	12.2%			Reference		16.5%			Reference	
Medium poverty	12.7%			1.074(1.004–1.149)	0.038	12.2%			1.232(1.028–1.478)	0.024
High poverty	10.4%			1.191(1.094–1.297)	< 0.001	10.5%			1.335(1.076–1.657)	0.009
Marital Status		138.625	< 0.001		< 0.001		12.351	0.006		0.005
Married	13.9%			Reference		13.0%			Reference	
Never married	13.4%			1.150(1.061–1.247)	0.001	14.7%			1.003(0.828–1.214)	0.977
Divorced/ Separated	10.2%			1.183(1.087–1.287)	< 0.001	13.3%			1.180(0.963–1.446)	0.111
Widowed	8.4%			1.179(1.104–1.260)	< 0.001	7.7%			1.379(1.143–1.664)	0.001

Abbreviations: SEER, Surveillance, Epidemiology, and End Results; CCS, cancer cause-specific survival; HR, hazard ratio; CI, confidence interval; NI, not included in the multivariate survival analysis.

*: Other includes American Indian/Alaska native, Asian/Pacific Islander, and unknown.

#: Because the record in the SEER database ended in 2013, the 5-year CCS of this group did not exist.

### Influence of marital status on ICSS

Although a better 5-year ICSS was observed in married individuals compared with unmarried individuals diagnosed with ICC (13.0% vs 11.6%) (Figure 1C), the log-rank χ2 test indicated that the difference was marginally significant (*P* = 0.056). After separating the unmarried status based on being never married, divorced/separated, and widowed, a substantial decrease in cancer-specific survival was observed in widowed patients compared to married patients (7.7% vs 13.0%, *P* = 0.006) (Figure 1D). After controlling other covariates using Cox regression model, it showed that the widowed status was an independent prognostic factor for poor survival outcome of ICC patients (HR 1.379, 95% CI 1.143-1.664, *P* = 0.001). Compared with married ICC patients, no significant difference was found in never married group (HR 1.003, 95% CI 0.828-1.214, *P* = 0.977) and divorced/separated group (HR 1.180, 95% CI 0.963-1.446, *P* = 0.111).

In addition, several covariates including gender (*P* = 0.016), age (*P* < 0.001), year of diagnosis (*P* < 0.001), pathological grading (*P* < 0.001), TNM stage (*P* < 0.001), SEER stage (*P* < 0.001) and socioeconomic status (*P* < 0.001) were proved as significant risk factors for ICC prognosis by univariate analysis (Table 2). Multivariate analysis was further carried out to identify the independent predictive factors, as follows: gender (female, HR 0.816, 95% CI 0.719-0.927), age (≥70, HR 1.276, 95% CI 1.114-1.461), year of diagnosis (1990-1999, HR 0.635, 95% CI 0.423-0.952), pathological grading (poor/anaplastic, HR 1.540, 95% CI 1.282-1.850), TNM stage (III/ IV, HR 1.535, 95% CI 1.217-1.938), SEER stage (regional, HR 1.236, 95% CI 1.025-1.491; distant, HR 1.852, 95% CI 1.523-2.252), and socioeconomic status (medium poverty, HR 1.232, 95% CI 1.028-1.478; high poverty, HR 1.335, 95% CI 1.076-1.657) (Table 2).

### Subgroup analysis of marital status on ECSS and ICSS according to SEER stage

**Figure 2:**
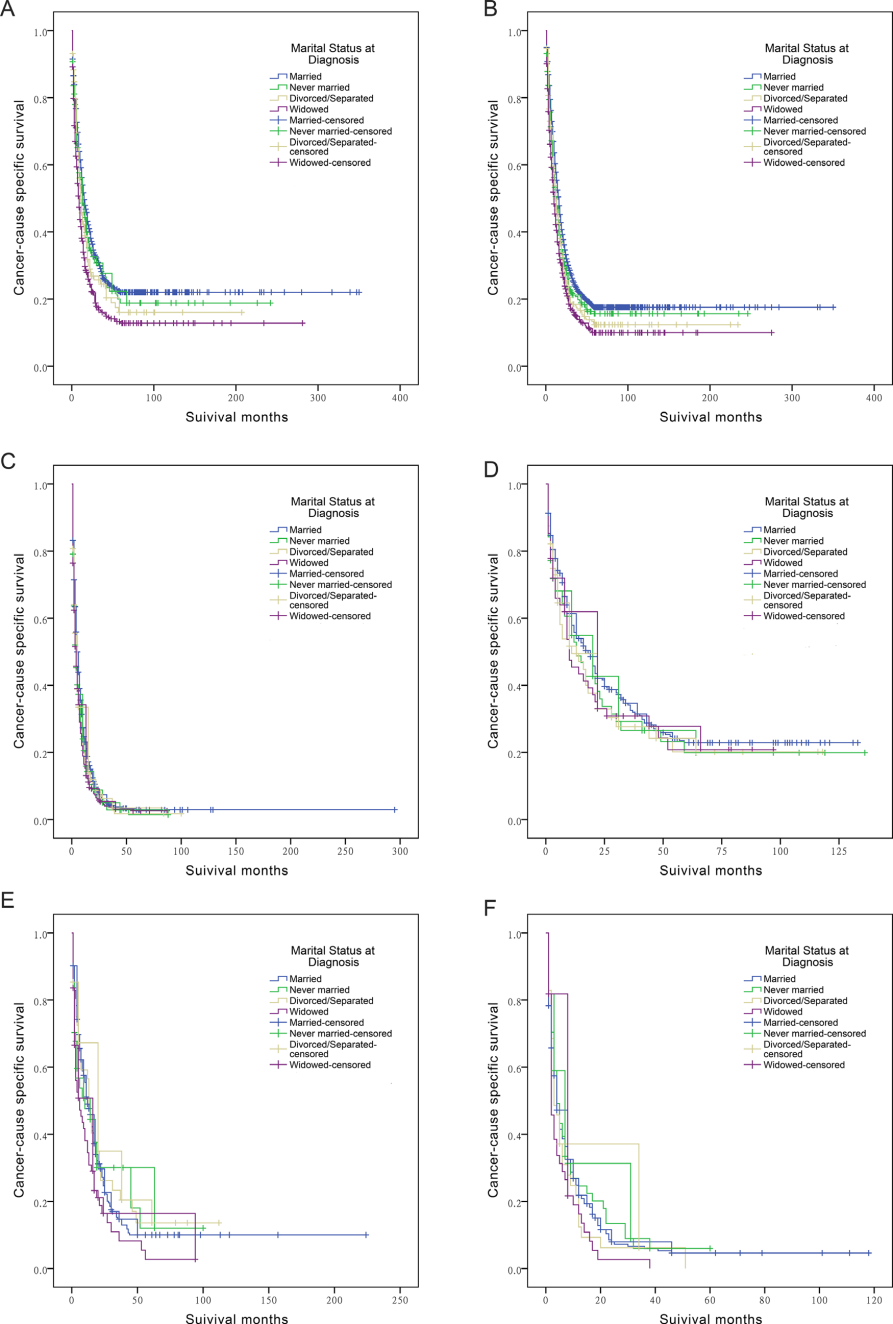
Survival curves in cholangiocarcinoma patients according to marital status (**A**) ECC patients at SEER localized stage: χ^2^ = 39.758 (*P* < 0.001); (**B**) ECC patients at SEER regional stage: χ^2^ = 54.452 (*P* < 0.001); (**C**) ECC patients at SEER distant stage: χ^2^ = 17.9 (*P* < 0.001); (**D**) ICC patients at SEER localized stage: χ^2^ = 2.05 (*P* = 0.56); (**E**) ICC patients at SEER regional stage: χ^2^ = 6.725 (*P* = 0.081); (**F**) ICC patients at SEER distant stage: χ^2^ = 5.054 (*P* = 0.168).

As shown in Table 3 and Figure 2A–2C, we assessed the effects of marital status on ECSS at each SEER stage. Univariate analysis showed that married ECC patients had the highest survival rate among all tumor stages: married patients had a 9.2% increase in 5-year ECSS compared with widowed patients for localized stage tumors (22.0% vs 12.8%), a 7.5% increase for regional stage tumors (17.5% vs 10.0%), and a 0.3% increase for distant stage tumors (2.9% vs 2.6%). Multivariate Cox regression analyses were carried out for different SEER stages. Marital status was validated as an independent predictor of ECC survival at localized stage (widowed, HR 1.196, 95% CI 1.043-1.372), regional stage (never married, HR 1.190, 95% CI 1.047-1.353; divorced/separated, HR 1.188, 95% CI 1.038-1.361; widowed, HR 1.124, 95% CI 1.004-1.257), and distant stage (never married, HR 1.237, 95% CI 1.066-1.435).

**Table 3:** Univariate and multivariate survival analysis of marital status on extrahepatic/intrahepatic cholangiocarcinoma cause-specific survival based on different SEER stages

Variable		Univariate analysis		Multivariate analysis	
	5-year CCS	Log rank χ2 test	*P*	HR (95% CI)	*P*
**Extrahepatic cholangiocarcinoma**					
**SEER stage**					
**Localized**					
**Marital Status**		39.758	< 0.001		0.034
Married	22.0%			Reference	
Never married	18.8%			1.165(0.954–1.423)	0.133
Divorced/Separated	16.0%			1.191(0.967–1.468)	0.1
Widowed	12.8%			1.196(1.043–1.372)	0.01
**Regional**					
**Marital Status**		54.452	< 0.001		0.004
Married	17.5%			Reference	
Never married	15.6%			1.190(1.047–1.353)	0.008
Divorced/Separated	12.3%			1.188(1.038–1.361)	0.013
Widowed	10.0%			1.124(1.004–1.257)	0.042
**Distant**					
**Marital Status**		17.9	< 0.001		0.008
Married	2.9%			Reference	
Never married	1.4%			1.237(1.066–1.435)	0.005
Divorced/Separated	1.7%			1.162(1.000–1.351)	0.05
Widowed	2.6%			1.134(0.997–1.289)	0.056
**Intrahepatic cholangiocarcinoma**					
**SEER stage**					
**Localized**					
**Marital Status**		2.06	0.56		NI
Married	22.9%				
Never married	19.9%				
Divorced/Separated	20.2%				
Widowed	20.8%				
**Regional**					
**Marital Status**		6.725	0.081		0.023
Married	10.0%			Reference	
Never married	12.0%			0.960(0.639–1.441)	0.843
Divorced/Separated	13.6%			1.070(0.727–1.574)	0.731
Widowed	2.7%			1.715(1.204–2.443)	0.003
**Distant**					
**Marital Status**		5.054	0.168		NI
Married	4.6%				
Never married	6.0%				
Divorced/Separated	0.0%				
Widowed	0.0%				

Abbreviations: SEER, Surveillance, Epidemiology, and End Results; CCS, cancer cause-specific survival; HR, hazard ratio; CI, confidence interval; NI, not included in the multivariate survival analysis.

In ICC patients, univariate and multivariate analysis demonstrated that widowed patients had a poorer outcome (HR 1.715, 95% CI 1.204-2.443) compared with married patients at regional stage, and no significant result was detected in any other subgroup (Table 3 and Figure 2D–2F).

### Subgroup analysis of marital status on ECSS and ICSS according to age at diagnosis

**Figure 3:**
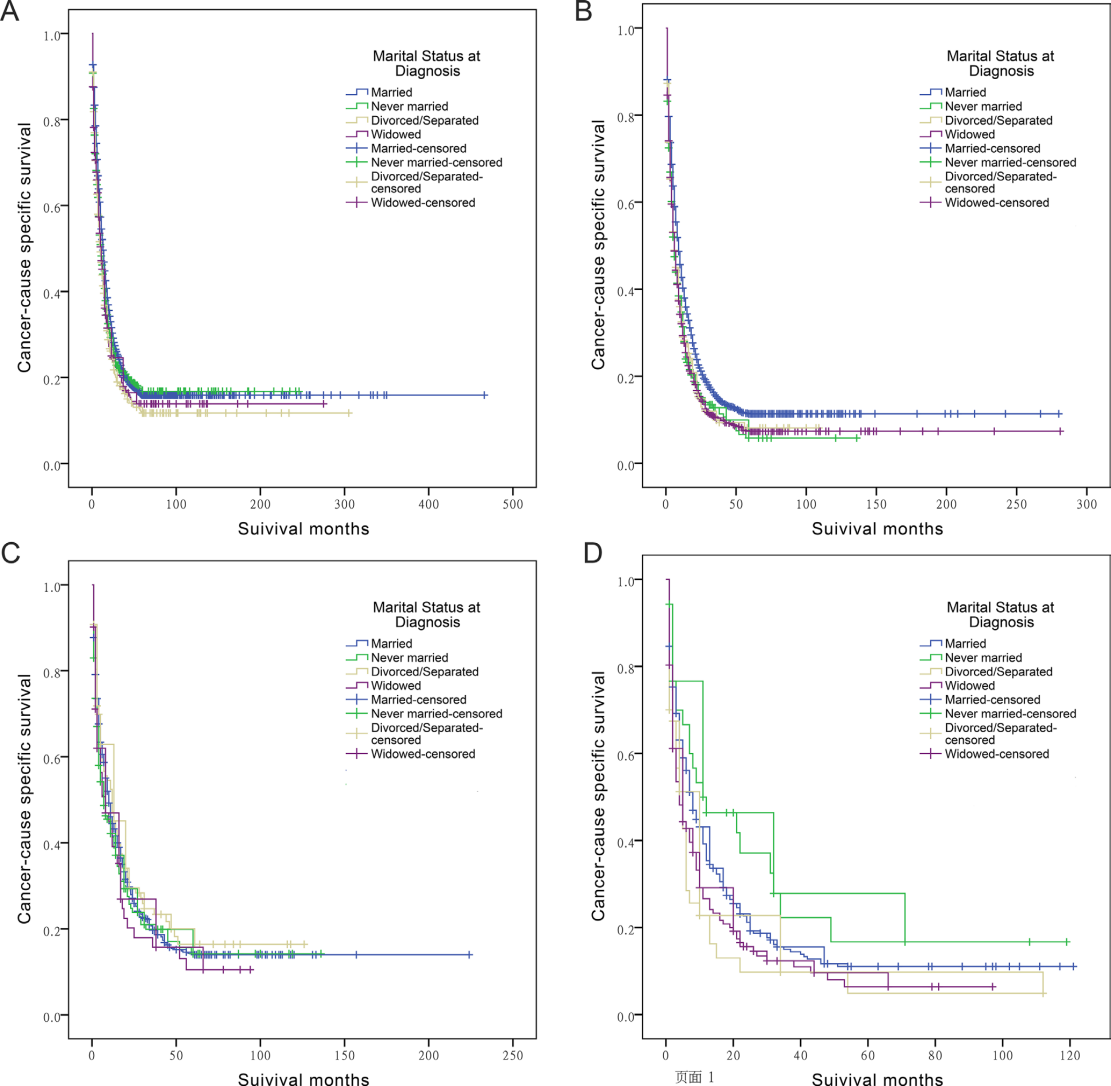
Survival curves in cholangiocarcinoma patients according to marital status (**A**) ECC patients aged less than 70: χ^2^ = 17.994 (*P* < 0.001); (**B**) ECC patients aged more than 70: χ^2^ = 57.184 (*P* < 0.001); (**C**) ICC patients aged less than 70: χ^2^ = 2.568 (*P* = 0.463); (**D**) ICC patients aged more than 70: χ^2^ = 13.195 (*P* = 0.004).

We further examined whether marital status was correlated to 5-year ECSS in patients diagnosed at different ages. Univariate analysis of marital status indicated that married patients had a better 5-year ECSS in all age subgroups (age < 70, 5-year ECSS 15.9%, *P* < 0.001; age ≥ 70, 5-year ECSS 11.4%, *P* < 0.001) (Table 4). Cox regression analysis confirmed the independent prognostic role of marital status in both younger patients (divorced/separated, HR 1.185, 95% CI 1.060-1.325) and older patients (never married, HR 1.258, 95% CI 1.104-1.434; divorced/separated HR 1.198, 95% CI 1.054-1.362; widowed HR 1.212, 95% CI 1.128-1.302) (Table 4 and Figure 3A–3B).

**Table 4:** Univariate and multivariate survival analysis of marital status on extrahepatic/intrahepatic cholangiocarcinoma cause-specific survival based on different age groups

Variable		Univariate analysis		Multivariate analysis	
	5-year CCS	Log rank χ2 test	*P*	HR (95% CI)	*P*
**Extrahepatic cholangiocarcinoma**					
**Age < 70**					
**Marital Status**		17.994	< 0.001		0.007
Married	15.9%			Reference	
Never married	16.8%			1.107(0.999–1.228)	0.053
Divorced/Separated	11.7%			1.185(1.060–1.325)	0.003
Widowed	13.9%			1.115(0.971–1.280)	0.124
**Age ≥ 70**					
**Marital Status**		57.184	< 0.001		< 0.001
Married	11.4%			Reference	
Never married	5.8%			1.258(1.104–1.434)	0.001
Divorced/Separated	8.1%			1.198(1.054–1.362)	0.006
Widowed	7.3%			1.212(1.128–1.302)	< 0.001
**Intrahepatic cholangiocarcinoma**					
**Age < 70**					NI
**Marital Status**		2.568	0.463		
Married	14.0%				
Never married	14.2%				
Divorced/Separated	16.4%				
Widowed	10.5%				
**Age ≥ 70**					
**Marital Status**		13.195	0.004		0.019
Married	11.0%			Reference	
Never married	16.7%			0.734(0.473–1.141)	0.169
Divorced/Separated	4.9%			1.339(0.925–1.938)	0.122
Widowed	6.4%			1.305(1.029–1.654)	0.028

Abbreviations: CCS, cancer cause-specific survival; HR, hazard ratio; CI, confidence interval; NI, not included in the multivariate survival analysis.

Univariate and multivariate survival analysis showed that widowed patients suffered from poorer survival outcomes (HR 1.305, 95% CI 1.029-1.654) compared to married patients when diagnosed at older age. In addition, there was no significant result observed in age < 70 subgroup (Table 4 and Figure 3C–3D).

## DISCUSSION

To the best of our knowledge, the current study is the first study to date comprehensively investigating the influence of marital status on the survival of patients with cholangiocarcinoma. We found a higher risk of death associated with being unmarried, especially widowed. Further subgroup analysis on SEER stage and age verified the prognostic value of marital status in cholangiocarcinoma.

Our results demonstrated that widowed cholangiocarcinoma patients were more likely to suffer from survival disadvantages. Marriage itself may increase the probability of early diagnosis. On most occasions, married patients experience more emotional and social support compared to those who are unmarried [19]. Married individuals are more likely to pursue immediate and aggressive treatment [20]. Besides, constant support provided by a spouse encourages the patients to comply with the prescribed treatment regiments [21, 22]. An alternative hypothesis is that although marriage itself may not necessarily protect the patients, once one has got married, becoming widowed is rather hard to endure both physically and psychologically. In consistent with this standpoint, no significant difference was found in our study between married patients and never married patients in ICC (HR 1.003, 95% CI 0.828-1.214, *P* = 0.977). Widowed patients, by contrast, had a poor survival outcome compared with married patients in ICC (HR 1.379, 95% CI 1.143-1.664, *P* = 0.001). It is conceivable that patients who are unmarried, especially the widowed ones, have a more fragile support network. The chronic stress that results from a lack of social support may impact cancer growth and metastasis by neuroendocrine mediators and cytokines [23]. A lack of psychological support leads to decreased activity of natural killer cells and increased mortality in cancer patients [24, 25].

An interesting observation was that the independent predictive value of marital status was more obvious in ECC patients than in those diagnosed with ICC. The never married, divorced/separated, and widowed patients all had poorer survival outcome than married patients in ECC, while only the widowed status independently predicted poor ICC survival. Subgroup analysis according to SEER stage and age indicated similar observations. These results suggested that the long-term prognosis of these two subtypes of cholangiocarcinoma was different, further confirming the heterogeneity between ECC and ICC.

In spite of our efforts to make an accurate and comprehensive analysis, some limitations of our analysis need to be addressed. First of all, the retrospective nature of the current study might result in bias and affect the results. Second, the SEER dataset only provides the marital status at diagnosis. Due to insufficient data, we could not describe or analyze information regarding changes in marital status, duration of the marriage, length of being single or the quality of marriage. Finally, cholangiocarcinoma predisposing factors (eg. primary sclerosing cholangitis, choledochal cysts, bile duct stones, inflammatory bowel disease, viral hepatitis, or infections with the liver fluke) were not provided in the SEER database. These confounding factors may potentially affect the results.

Despite these potential limitations, the present study was based on a large population and multiple centers, and is therefore reliable and convincing. We separated unmarried participants based on being never married, divorced/separated, and widowed, and each SEER stage and age group was individually investigated with a variety of traditional risk factors taken into consideration. Our results confirmed the independent prognostic effect of the unmarried status with a varied risk compared to the married status. Furthermore, the results demonstrated that the unmarried patients were heterogeneous, and widowed patients tended to be at the highest risk of cancer cause-specific death compared to those in other groups. Social support, psychological factors and advanced tumor stage might be the reasons for survival disadvantages in widowed patients. More social care and support should be provided for the unmarried patients, especially the widowed ones.

## MATERIALS AND METHODS

### Patient selection in the SEER dataset

All primary data were obtained by using SEER*Stat software version 8.3.2 and the SEER database released in April 2016. The SEER dataset contains no identifiers, and has been widely used for studies examining the association between marital status and cancer survival [26–31]. County-level socioeconomic information for the year 2000 was obtained from US Census 2000 files, which was made available by the US Census Bureau and linked to the SEER database.

ECCs were identified by the topography code C24.0 for extrahepatic bile duct with the following morphology codes: 8010, 8020, 8041, 8070, 8140, 8144, 8160, 8161, 8260, 8310, 8480, 8490, 8560 and 8162. ICCs were defined by topography code C22.0 for liver and morphology codes 8160 and 8161, or by topography code C22.1 (intrahepatic bile duct) and morphology codes 8010, 8020, 8140, 8160 and 8161, as previously reported [32].

The exclusion criteria included: (1) patients with ECC or ICC identified only by autopsy or death certificate, (2) patients had more than one primary tumor but ECC/ICC was not the first one, (3) age at diagnosis was less than 18, (4) unknown survival time or survival time of 0 months, (5) unknown marital status, (6) SEER stage *in situ* or unknown.

The TNM stage was established according to the criteria described in the American Joint Committee on Cancer (AJCC) Cancer Staging Manual (the 6^th^ edition). Socioeconomic status was determined by the county poverty rate [33, 34], which was defined as the percentage of people in the county living below the national poverty threshold in the 2000 US Census. As previously reported, the county poverty rates were categorized into three levels using the same cut points used in the National Cancer Institute monograph: < 10% (low-poverty areas), 10%–19.99% (medium-poverty areas), and ≥ 20% (high-poverty areas) [35].

### Statistical analysis

Differences in baseline characteristics were compared by Pearson chi-squared test for categorical variables. ECSS and ICSS were calculated by the Kaplan-Meier method and log-rank χ2 tests were conducted to compare differences between subgroups of each variable. Multivariate Cox proportional hazard models were adopted to determine risk factors that might affect survivorship. All *P* values were two-sided, and *P* < 0.05 was regarded as statistically significant. All data were analyzed by SPSS version 21.0 (Statistics Package for Social Science, Chicago, IL).

## SUPPLEMENTARY MATERIALS TABLES



## Abbreviations

ECC: extrahepatic cholangiocarcinoma
ICC: intrahepatic cholangiocarcinoma
SEER: Surveillance, Epidemiology, and End Results
HCC: hepatocellular carcinoma
ECSS: extrahepatic cholangiocarcinoma cause-specific survival
ICSS: intrahepatic cholangiocarcinoma cause-specific survival
HR: hazard ratio
CI: confidence interval
AJCC: American Joint Committee on Cancer.
