# Supplementary material for: Influence of marital status on the survival of adults with extrahepatic/intrahepatic cholangiocarcinoma
Source: Oncotarget. 2017 Mar 17;8(17):28959–70. doi: 10.18632/oncotarget.16330 (PMC5438705; doi:10.18632/oncotarget.16330)
Supplement: Supplementary file 1 [file oncotarget-08-28959-s001.pdf]

Influence of marital status on the survival of adults with extrahepatic/intrahepatic cholangiocarcinoma

Supplementary Materials

Supplementary Table 1: Baseline demographic and cancer characteristics of extrahepatic cholangiocarcinoma patients in SEER database

Characteristic	Total	Married	Never married	Divorced/ Separated	Widowed	*P*
	(*n* = 8776) N(%)	(*n* = 5096) N(%)	(*n* = 974) N(%)	(*n* = 799) N(%)	(*n* = 1907) N(%)	
Gender						< 0.001
Male	4590(52.3)	3272(64.2)	527(54.1)	395(49.4)	396(20.8)	
Female	4186(47.7)	1824(35.8)	447(45.9)	404(50.6)	1511(79.2)	
Age						< 0.001
< 70	4179(47.6)	2762(54.2)	641(65.8)	469(58.7)	307(16.1)	
≥ 70	4597(52.4)	2334(45.8)	333(34.2)	330(41.3)	1600(83.9)	
Ethnicity						< 0.001
White	6867(78.2)	4033(79.1)	699(71.8)	642(80.4)	1493(78.3)	
Black	706(8.0)	291(5.7)	147(15.1)	87(10.9)	181(9.5)	
Other*	1203(13.7)	772(15.1)	128(13.1)	70(8.8)	233(12.2)	
Year of diagnosis						< 0.001
1973–1979	446(5.1)	305(6.0)	15(1.5)	48(6.0)	78(4.1)	
1980–1989	701(8.0)	389(7.6)	61(6.3)	75(9.4)	176(9.2)	
1990–1999	1088(12.4)	633(12.4)	123(12.6)	80(10.0)	252(13.2)	
2000–2009	4294(48.9)	2434(47.8)	481(49.4)	382(47.8)	997(52.3)	
2010–2013	2247(25.6)	1335(26.2)	294(30.2)	214(26.8)	404(21.2)	
Pathological grading						< 0.001
Well/Moderate	2447(27.9)	1597(31.3)	260(26.7)	207(25.9)	383(20.1)	
Poor/Anaplastic	1394(15.9)	870(17.1)	145(14.9)	146(18.3)	233(12.2)	
Unknown	4935(56.2)	2629(51.6)	569(58.4)	446(55.8)	1291(67.7)	
TNM Stage						< 0.001
I/II	2396(27.3)	1463(28.7)	271(27.8)	221(27.7)	441(23.1)	
III/IV	1785(20.3)	1063(20.9)	241(24.7)	187(23.4)	294(15.4)	
Unknown	4595(52.4)	2570(50.4)	462(47.4)	391(48.9)	1172(61.5)	
SEER Stage						< 0.001
Localized	1738(19.8)	974(19.1)	173(17.8)	146(18.3)	445(23.3)	
Regional	3589(40.9)	2258(44.3)	407(41.8)	318(39.8)	606(31.8)	
Distant	2085(23.8)	1250(24.5)	244(25.1)	218(27.3)	373(19.6)	
Unstaged	1364(15.5)	614(12.0)	150(15.4)	117(14.6)	483(25.3)	
Socioeconomic Status						< 0.001
Low poverty	1373(15.6)	845(16.6)	122(12.5)	119(14.9)	287(15.0)	
Medium poverty	5969(68.0)	3478(68.2)	695(71.4)	555(69.5)	1241(65.1)	
High poverty	1434(16.3)	773(15.2)	157(16.1)	125(15.6)	379(19.9)	

Abbreviations: SEER, Surveillance, Epidemiology, and End Results.
*Other includes American Indian/Alaska native, Asian/Pacific Islander, and unknown.

Supplementary Table 2: Baseline demographic and cancer characteristics of intrahepatic cholangiocarcinoma patients in SEER database

Characteristic	Total	Married	Never married	Divorced/ Separated	Widowed	*P*
	(*n* = 1352) N(%)	(*n* = 830) N(%)	(*n* = 176) N(%)	(*n* = 148) N(%)	(*n* = 198) N(%)	
Gender						< 0.001
Male	675(49.9)	472(56.9)	102(58.0)	63(42.6)	38(19.2)	
Female	677(50.1)	358(43.1)	74(42.0)	85(57.4)	160(80.8)	
Age						< 0.001
< 70	880(65.1)	570(68.7)	141(80.1)	108(73.0)	61(30.8)	
≥ 70	472(34.9)	260(31.3)	35(19.9)	40(27.0)	137(69.2)	
Ethnicity						< 0.001
White	1102(81.5)	692(83.4)	129(73.3)	116(78.4)	165(83.3)	
Black	129(9.5)	46(5.5)	39(22.2)	24(16.2)	20(10.1)	
Other*	121(8.9)	92(11.1)	8(4.5)	8(5.4)	13(6.6)	
Year of diagnosis						0.832
1973–1979	50(3.7)	36(4.3)	4(2.3)	4(2.7)	6(3.0)	
1980–1989	84(6.2)	56(6.7)	8(4.5)	5(3.4)	15(7.6)	
1990–1999	61(4.5)	37(4.5)	8(4.5)	7(4.7)	9(4.5)	
2000–2009	780(57.7)	477(57.5)	103(58.5)	86(58.1)	114(57.6)	
2010–2013	377(27.9)	224(27)	53(30.1)	46(31.1)	54(27.3)	
Pathological grading						0.239
Well/Moderate	379(28.0)	242(29.2)	48(27.3)	44(29.7)	45(22.7)	
Poor/Anaplastic	276(20.4)	174(21.0)	28(15.9)	34(23.0)	40(20.2)	
Unknown	697(51.6)	414(49.9)	100(56.8)	70(47.3)	113(57.1)	
TNM Stage						0.004
I/II	268(19.8)	151(18.2)	35(19.9)	42(28.4)	40(20.2)	
III/IV	471(34.8)	294(35.4)	65(36.9)	58(39.2)	54(27.3)	
Unknown	613(45.3)	385(46.4)	76(43.2)	48(32.4)	104(52.5)	
SEER Stage						0.008
Localized	443(32.8)	276(33.3)	57(32.4)	56(37.8)	54(27.3)	
Regional	389(28.8)	246(29.6)	41(23.3)	41(27.7)	61(30.8)	
Distant	360(26.6)	226(27.2)	55(31.3)	35(23.6)	44(22.2)	
Unstaged	160(11.8)	82(9.9)	23(13.1)	16(10.8)	39(19.7)	
Socioeconomic Status						0.254
Low poverty	197(14.6)	121(14.6)	24(13.6)	20(13.5)	32(16.2)	
Medium poverty	899(66.5)	570(68.7)	111(63.1)	96(64.9)	122(61.6)	
High poverty	256(18.9)	139(16.7)	41(23.3)	32(21.6)	44(22.2)	

Abbreviations: SEER, Surveillance, Epidemiology, and End Results.
*Other includes American Indian/Alaska native, Asian/Pacific Islander, and unknown.
